# Post Disaster Governance, Complexity and Network Theory: Evidence from Aceh, Indonesia After the Indian Ocean Tsunami 2004

**DOI:** 10.1371/4f7972ecec1b6

**Published:** 2015-07-01

**Authors:** Jonatan A. Lassa

**Affiliations:** Nanyang Technological University

## Abstract

This research aims to understand the organizational network typology of large­-scale disaster intervention in developing countries and to understand the complexity of post-­disaster intervention, through the use of network theory based on empirical data from post-­tsunami reconstruction in Aceh, Indonesia, during 2005/­2007. The findings suggest that the ‘ degrees of separation’ (or network diameter) between any two organizations in the field is 5, thus reflecting ‘small­ world’ realities and therefore making no significant difference with the real human networks, as found in previous experiments. There are also significant loops in the network reflecting the fact that some actors tend to not cooperate, which challenges post­ disaster coordination. The findings show the landscape of humanitarian actors is not randomly distributed. Many actors were connected to each other through certain hubs, while hundreds of actors make ‘scattered’ single ‘principal-­client’ links. The paper concludes that by understanding the distribution of degree, centrality, ‘degrees of separation’ and visualization of the network, authorities can improve their understanding of the realities of coordination, from macro to micro scales.

## 1. Introduction

Large scale disasters occur in both developed and developing countries. Hurricane Katrina in 2005 in the United States and the Tōhoku earthquake and tsunami in 2011 in Japan are examples of large natural catastrophic event in the 21st century that occurred in developed countries. More large scale natural catastrophes in this century have occurred in developing countries such as Indian Ocean Tsunami that hit 14 countries in Indian Ocean and West Africa in 2004, Cyclone Nargis in Myanmar in 2008, devastating earthquakes in Haiti in 2010 and Nepal earthquakes in 2015.

Developed countries seem to be more independent in dealing with large scale disasters. Within the context of developing countries, it has been observed that major catastrophes trigger the requirement for external organizations to come in and help the survivors. The involvement of hundreds to thousands of non­state and non­governmental actors after big catastrophes in these countries may create more complex realities beyond the comprehension and the capacity of the respective actors such as governments and the local disaster response authority. Recent large-­scale disasters in Asia (e.g. Indonesia, Myanmar, Pakistan) and the Caribbean (Haiti) resulted in high involvement by international non­governmental organizations and international organizations (INGOs/IOs)

In Southeast Asia large-­scale disasters and the presence of INGOs/IOs then trigger the creation of hundreds to thousands of local NGOs.^1^^,^ 2 The author observed the rise of NGOs where large-­scale crisis events took place in Indonesia after the Indian Ocean Tsunamis in 2004 and Cyclone Nargis in Myanmar in 2008. Stumpenhorst et al.^1^ found that following the 2010 earthquake in Haiti, the number of NGOs increased uncontrollably. Admitting that large catastrophes create complex issues is just the beginning of solving the problem. A lack of understanding of the complexity landscape creates coordination problems, such as the over concentration and overlapping of intervention only in some areas. Available data suggest more than 1000 local, national and international organizations (including state and non­state actors) delivered their post­-disaster intervention in at least 5000 multi­sector projects post Indian Ocean Tsunami (IOT) in Aceh. Recent large-­scale disasters also show similar trends in terms of involvement of actors. More than 500 INGOs/IOs were involved post Cyclone Nargis in Myanmar 2008 and more than 700 INGOs/IOs responded to devastating earthquakes in Haiti 2010. These figures (Myanmar and Haiti) do not yet include local and national organizations.^5^

Proper coordination can foster better aid efficiency and effectiveness in post-­disaster settings. Unfortunately, given the complexity of response to large scale disasters, that are often chaotic and uncoordinated, it is difficult to achieve efficiency and effectiveness. The situation can sometime lead to a ‘tragedy of the commons’.^3^ As illustrated by the American ecologist, Garrett Hardin, the ‘tragedy of the commons’ is the use of common property resources where limited natural resources are exploited by local individuals and households without the intent to destroy local sustainability – but, unfortunately, in the end everybody loses. Following the IOT, especially in Aceh during 2005­-2007, the author observed another ‘tragedy of the commons’ phenomenon where the commons were humanitarian and reconstruction aid. In this ‘game’, the commons are expected to be extinct when the reconstruction resources end. In the context of humanitarian aid, aid players expect aid to be stopped after a certain period of time. Both aid players and survivors can be losers. But the ‘game’ will be repeated in new disaster areas where similar INGOs/IOs are likely to exercise their ‘moral imperative’ with already limited aid resources. In the end, how could everybody win in this game to meet the vision of rebuilding resilient (social/­physical) structures that can better absorb future shocks? In this paper, the author argues that without understanding the complex network of post-­disaster intervention, both the aid players and the survivors will end as losers.

Unfortunately, there is still lack of academic endeavor to use network theory for disaster research not only outside US and European contexts, but also for large-­scale emergencies worldwide. In addition, the use of the approach in the US context is limited to a much smaller scale of nodes (organizational actors) involved in Katrina. This paper shows a disaster governance setting from developing countries, by focusing on the Indian Ocean Tsunami of 2004 in Aceh and Nias, Indonesia, where a “big ­bang” formation of post-­disaster networks took place during 2005-­2007. It also provides evidence concerning the network typology of large organizational networks following a large­scale disaster.

This paper hypothesizes that understanding complexity through the use of network theory can help improve the performance of post­-disaster interventions, especially in the context of large-­scale natural hazards. The author uses the case of the Indian Ocean Tsunami 2004 in Aceh to demonstrate the potential use of network theory to unpack the complexity of aid agencies and organizations in post-disaster situations.

The key questions include: what does the complexity landscape for a typical network of humanitarian aid for large-­scale disasters look like? What does it mean for managing complexity in post-­disaster governance?

Section 2 discusses how network theory can be used to understand the details of complexity of organization-­to-­organization coordination. Section 3 discusses the concept of polycentric governance and its connection with network theory. Section 4 describes the research method. Section 5 provides the findings, which will be discussed in Section 6. Closing remarks are provided in Section 7.

## 2. Large Scale Disasters and Complexity

Post­-disaster intervention in Aceh and Nias (Indonesia) is complex.^2^ The recorded disaster mortality in Aceh due to the Indian Ocean Tsunami in 2004 is about 170,000. Post­-disaster interventions were exacerbated by the legacy of 30 years of civil war in the region. A rather successful peace process that later led to a more conducive situation added weight to complexity of post­-war and post-­disaster recovery. Based on the author’s direct experience as a field worker during reconstruction in Aceh after the 2004 Indian Ocean Tsunami, it was seen that a high asymmetry of information could lead to unnecessary and unhealthy competition by aid players. This often led to a situation where five INGOs and contractors competed to lobby the same local communities to offer housing aid. At worst, three INGOs/IOs could end up building houses in the same village, using different legitimacy approaches: one INGO made a deal with villagers directly, while the other two separately dealt with and gained permission from district governments and the reconstruction authority. The challenge was that there was no clear authority existed especially in the aftermath of large scale disasters. Conventional methods that guide understanding of post-­disaster complexity proved ineffective. It took a longer time to understand the macro picture of reconstruction players’ behaviors. Unfortunately, by the time the reconstruction authority began to understand the actors in more detail , the reconstruction period (often politically determined by national regulation) might already be ending. Experienced and trained authorities and officials are often struggling to deal with post­-disaster complexity because they have rarely experienced a similar scale of disaster before. The quality of field intervention therefore always suffers from the lack of comprehension of the multifaceted problems of the field.

Large scale disasters often created a breakdown in local institutions and governments leading to lack of clarity of authorities on the ground. This paper argues that conventional methods to guide understanding of post­disaster complexity proved ineffective. It took a longer time for local authorities to understand the macro picture of reconstruction players’ behaviors. Unfortunately, by the time the reconstruction authority began to understand the actors in more detail, the reconstruction period (often politically determined by national regulation) might already be ending. Experienced and trained authorities and officials are often struggling to deal with post­disaster complexity because they have rarely experienced a similar scale of disaster before. The quality of field intervention therefore always suffers from the lack of comprehension of the multifaceted problems on the field.

Complexity is now understood as one of the features of post­disaster reconstruction situations, which makes coordination difficult. Boin et al. argued that “coordination is the Holy Grail of disaster response: the call for more and better coordination is heard during and after most disasters. How complex networks under disaster conditions can be orchestrated remains unclear at best, however.”^4^ Humanitarian coordination is a tool that is used to achieve organized behaviors to produce desired outcomes such as effectiveness, efficiency and accountability in disaster responses. Coordination is difficult because existing aid bureaucrats often use irrelevant metrics and tools to understand the complex situations.

Large­scale disasters or major catastrophes can be defined as events that trigger the loss of lives in the hundreds to thousands, and that affect millions of people, collapse/damage thousands of buildings and create huge economic losses in proportion to the scale of economy of the areas affected. They create complexity that often goes beyond the comprehension of local authorities.

Intergovernmental and interorganizational interaction in a disaster context is complex.^6^ Large­scale disasters can hypothetically trigger the new formation of actor networks, such as global­local humanitarian actors. In the context of developing countries, post­disaster governance is arguably more complex due to a lack of human resources and high information asymmetry (the result of a dysfunctional infrastructure/lack of communication following a catastrophe, and a lack of transparency and information sharing). This invites external actors to come and conduct their humanitarian imperatives.

Comfort et al.^7^ promote the usefulness of network theory in the context of disaster coordination and response systems. Butts et al.^8^ has provided good examples of actors’ communication networks in the World Trade Center disaster. Kapucu^9^ demonstrates an early use of network theory in understanding multiorganizational communication and coordination in a disaster context at a smaller scale. Informed by their network analysis, Kapucu et al.^6^ found that, in the US, effective post­disaster intervention comes from bottom­up and local organizations, who are usually fast and more responsive to disasters.

Varda and colleagues^10^ noted the use of social network methods in disaster studies based on a post­ Katrina context, by assessing the network of the socially isolated groups. The Katrina disaster in 2005 also triggered new opportunities for US­based scientists to explore the use of network theory in understanding post­disaster interventions. Magsino^11^ recently reported on an initiative from the National Academy of Sciences to explore the use of network analysis to inform national disaster management planning.

Creating a centralist incident command system and structure for post­disaster intervention is a serious challenge; especially when higher­level authority can barely understand the landscape of complexity. Even though there may be options to suggest more decentralized intervention systems, such as the humanitarian cluster system recently promoted in global humanitarian response systems (see Table 1), such efforts may miss other emerging (uncontrolled) clusters that may not fit into the traditional sense of sectors and the humanitarian cluster system.

**Table 1. Humanitarian emergency cluster and cluster convenors:** Adapted from Stumpenhorst et. al.1 and Stoddard et. al.14

Name of cluster	Convenor or cluster leader	Remarks/web links
Water, Sanitiation and Hygiene (WASH)	United Nations International a Children's Emergency Fund (UNICEF)	At subnational level, lead cluster can be a cluster member. See WASH clustermembers at http://washcluster.net/
Education	UNICEF and Save the Children Alliance	http://logcluster.org
Agriculture	Food and Agriculture Organization (FAO)	
Health	WHO (World Health Organization)	
Emergency Shelter	United Nations High Commissioner for Refugees (UNHCR) (for conflict) and International Federation of Red Cross and Red Crescent (IFRC) (for natural hazards)	http://sheltercluster.org
Early Recovery	UNDP (United Nations Development Programme)	http://www.earlyrecovery.info
Camp coordination and management	UNHCR and IOM (International Organisation for Migration)	In Haiti, only IOM. See www.globalcccmcluster.org
Logistics	WFP (World Food Programme)	http://logcluster.org

## 3. Polycentric disaster governance and complex networks

A disaster­-risk governance framework recognizes the polycentric nature of disaster risk and emergency management, where there are many overlapping arenas (or centers) of authority and responsibility for disaster­-risk reduction and post-­disaster intervention. In this paper, polycentric governance refers to the nature of decision making in humanitarian emergencies as functioning across many centers and domains and across scales and levels.^12^^,^ 13 Evidence of polycentric governance also appears in the context of emergency management today, especially under the concept of the humanitarian cluster approach (see Table 1), as currently promoted by international actors. Under network theory, the ‘many centers’ emerge as hubs and sub-­networks of interorganizational actors.

Experienced field workers and specialists of international humanitarian emergencies may have predicted that the convenors of humanitarian clusters (Table 1) are the ones that are most likely to have high connections with regard to post disaster organizations’ networks. The cluster convenors are most likely to be part of the centers or hubs, while some other local organizations may hypothetically be the actors in the periphery. For instance, the International Federation of the Red Cross (IFRC) is likely to be a hub in the network because it is mandated to be the lead or convenor of emergency shelter clusters. Likewise, UNDP (United Nations Development Program) is likely to be important because it is mandated to lead early recovery clusters, which interact with most of the cluster leads. Table 1. Humanitarian emergency cluster and cluster convenors Source: Adapted from Stumpenhorst et. al.1 and Stoddard et. al.^14^ The humanitarian cluster approach is basically a means for coordinating clustered actors who have different responsibilities during humanitarian relief emergency responses, such as for agriculture, health and emergency shelter clusters.1 Each organization can sign up for more than one cluster membership. Big organizations may sign up for more than five cluster memberships. The lead of each cluster is listed in Table 1. However, most actors do not comply with the cluster approach, which in practice can create the overlapping of aid and conflict over aid on the field.

A network (or networked) governance model challenges the old assumption of structural analysis in social science (including economics and engineering) that disaster management outcomes simply arise from the sum of efforts from agents, namely, individuals and organizations.^12^^,^ 13 When it comes to analysis, disaster researchers tend to believe in the aggregation of variables and sums of actors. Applied network theory advocates the fact that agents and institutions exist and co­exist more in the form of networks. Furthermore, not simply a network approach versus aggregation, the theory asks: What kinds of networks are we actually dealing with? This argument is based on the emerging form of governance as networks of individuals and organizations/institutions (see Jones et al.^15^, Stocker^16^ and Crawford^17^ ). In the study of governments, Goldsmith and Eggers noted growing spaces where governments purposefully network with other networks of providers (of public goods) to enhance the delivery of public goods to meet their policy goals.^18^ The defined networks could involve third-­party government, that is, private firms and NGOs, or joined-­up government in the form of multiple and multilevel government agencies.

## 4. Research Methods

Network theory stems from graph theory, a branch of mathematics. The network theory suggests that it is not the sums of parts that matters but the connection of parts that matters most.^19^ Castells defines a network as a set of interlinked nodes, or, a node is the point at which a link intersects itself.^20^ A node can be an organization or an individual in a particular situation. A social network is a social structure made of agents that are coded as nodes that are tied with other agents (nodes, also known as vertices).^21^ Quantitative sociologists turn graph theory into social network analysis (SNA) to analyze ties among people, groups of people, organizations, and countries. Together, these ties form networks. Hence, SNA detects and interprets patterns of social ties among actors (see Nooy, Mrvar and Batagelj).^22^

Social network theorists argue that network analysis presents a better explanation of social behavior because it assumes a society is by no means merely a sum of individuals – instead, society is actually comprised of networks of individuals, organizations, and institutions. The network is also known as a graph. A graph is a set of nodes and a set of lines between pairs of nodes. A graph represents the structure of a network; all it needs for this is a set of nodes (or vertices/points) and a set of lines (links) where each line connects two vertices. A line connects two dots or endpoints or vertices (nodes).

A node is the smallest unit in a network and can represent either an agent (e.g., an organization, an adult female/male, a biological cell, an object) or an institution Furthermore, a node/vertex can be identified by a number or a label. A line connects two nodes in a network, which can represent any relational quality. Loops are important to note because they represent organizations or actors that may not be linked with others and only represent themselves. They could be generous private agencies, for example, that come and distribute whatever forms of aid they are providing without being connected to the existing humanitarian cluster system. In the network structure, they must appear as standalone actors. The diameter of network and the average path length of the networks and loops will be measured. The distance is measured by the number of links for one node to connect to other node. The diameter of a network is the largest distance between any two nodes in the network. The average path length is the average distance between any two nodes in the network – a measure of efficiency of transmitting information or ideas. The later variable is bounded, but can be much shorter than by the former variable. Two types of centrality analysis are used, namely *degree* centrality and *betweenness* centrality. Centrality analysis refers to positions of individual vertices (or nodes) within a network. *Degree* centrality is the easiest to measure as it is the number of ties (or links) connected to a given node, or “the number of nodes that the focal node is connected to”.^23^ To determine the leader(s) in a network of inter­organization (to represent the lead institution/ organization or individual leader of a unit of community or set of organisations), one can identify the highest value of* betweenness* centrality.^22^^,^ 24 Nooy, Mrvar, and Batagelj^22^ argue that the more a node is a go-­between, the more central its position in the network. It means that the more a node possesses dense relational ties between other nodes (agents/actors and/or organizations), the more important the node is to the flow of any aid resource in the post­-disaster reconstruction network.

The *betweenness* centrality has a value between 0 and 1. The higher the value, the higher the centrality of the node in the network, which is an indication of leadership or a hub function. Since a post-­disaster interventions network involves ‘donor-­partner’ relationships, two measures will be introduced: the first is the in-degree, measured by the number of links (arcs) a node receives. In-degree is therefore first-­level partners or ‘implementing partners’. The second is the out-degree, measured by the number of links (arcs) it sends (e.g. the number of organizations a donor transfers grants to). In this context, it is the donor or the direct source of the first-­level partners. Therefore, these two measures must recognize the direction of links. In the case where organizations use their own resources to distribute aid (without intermediaries), it is called self-­sponsorship and, therefore, is considered a self­ transaction which is seen as a solitaire node (unconnected to the rest of the actors) or loop. However, such solitaire nodes are all treated as part of the network because of their willingness to report their activities to the existing reconstruction authority. Visually, a self-­sponsorship organization appears as one unconnected node.

This paper also evaluates the k­-core of the network. A k-­core classifies relatively dense sub-­networks to find cohesive subgroups. “A k-­core is a maximal sub­network in which each vertex (node) has at least *degree* k within the sub-network.” K­-core is used to identify clusters of nodes that are tightly connected because each node has a “particular minimum *degree* within the cluster”.^21^^,^ 22 ^,^ 23 A 2-­core or 3-­core network means all nodes that are connected by *degree* subsequently two more or three more to other nodes within the core network. Data Source. The data used in this analysis is derived from April 2007, updated by the Aceh­-Nias Rehabilitation and Reconstruction Agency (known as BRR). This is the latest version accessible to the author. The author often contributed to BRR project updates using an online system which no longer exists. The updates are often distributed to international agencies and INGOs. Even though it is not the final version (as BRR mandates ended in 2009), there is enough information to analyze the network properties. The spread sheet consists of “financial updates” – different project financing updates from more than 800 different actors (donors and implementing partners). The updates contain 1300 project financing updates broken down into 5000 different project outputs. Any organization A can have more than two transactions (and more project outputs) supported by organization B in more than one different sector. Due to time limitations, the analysis focuses on the donor­-partner data. Therefore, regardless of how many projects and the size of the contracted projects made between any two nodes (principal­-client or donor-­partner), it will be treated as one single link. This approach does not discount data quality in terms of the formal links between the organizations. After data cleaning, the data-set has a total of 797 organizations; 797 organizations means 797 nodes. Software. For the overall analysis of the network of post-­disaster governance in Aceh, the author used Pajek’s algorithm – a detailed explanation can be found in Nooy, Mrvar, and Batagelj^22^ . Gephi’s algorithm is used as an alternative visualization for good qualitative interpretation.

## 5. Results: Visualization of Post Disaster Actor Network

Based on both Gephi’s network analysis and Pajek’s network algorithm, the diameter of the network is 5 (see Figure 1, Figure 2A and Figure 2B), with n = 797 nodes and 977 total links. The average path length is 1.715 (based on Gephi). The number of loops is 28, meaning that there are 28 nodes that link only to themselves. These loops are visible in Figure 2A.

Ten categories (partitions) were made: Aceh-­Nias Reconstruction and Rehabilitation Agency (BRR Aceh­ Nias); Indonesia government institutions at a national level; local government organizations; bilateral aid from independent countries; multilateral aid organizations such as the United Nations, including the World Bank; international NGOs; local­-national NGOs; private firms; universities; and others (none of the above). There were 472 INGOs in Aceh and Nias during 2005­/2007 (Table 2) delivering their post­ disaster reconstruction aid (from housing to agricultural to health and other sectors). There were 147 NGOs. There were 25 multilateral organizations (such as United Nations agencies UNDP, WFP), including the World Bank and European Commission. There were 36 bilateral donors involved in this analysis (including the Australian Government, US Government, French and German governments, and so on). The Aceh­-Nias Rehabilitation and Reconstruction Agency is grouped alone as BRR (Table 2). BRR is a multi­sector agency as it involved with and governs all the reconstruction sectors. Table 2. Sums of *degree, outdegree* and *indegree* Source: Author. Data from BRR April 2007. The calculation uses Pajek mode 1 (a directed network).

**Visualized Networks of Organizational Post Disasters Intervention 2005-­2007:**
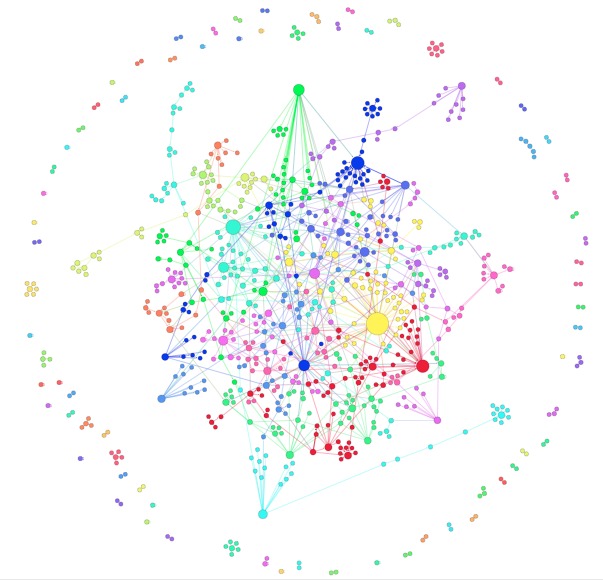
Based on Force Atlas layout in Gephi. The size of the nodes reflect the degree of the nodes. The colors of the nodes reflect 90 communities detected within the network. The number of communities suggest the relative (un)connectedness of the dots (organisations). The nodes with low connection are scattered in outer boundary or periphery.

**Visualized Networks of based on degree , betweeness and k-core:**
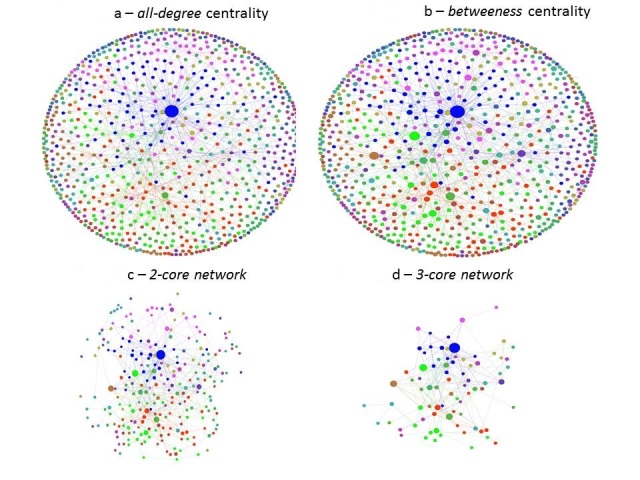
Figure 2A visualizes the all-­degree ( or degree ) network (based on the number of links each node possesses. Figure 2B shows the centrality of actors (or ‘leadership’ of each node within the network. Figure 2C and 2D are k-­core networks, which mean all nodes that are connected by k degree (or links) (or in this case 2 and 3 subsequently). The Figures are base don Gephi's ARF layout.

**Degree, Indegree and Outdegree Analysis**

Figure 2A and 2B visualise the difference between *degree* centrality (possession of links) and *betweeness* centrality (a measure of a more strategic position or influence). Figure 2A and Table 3 show that 1.62% of nodes (17 organizations) are linked to more than 15 nodes. The highest connected node is UNDP (degree centrality). UNDP also possesses the highest *betweenness* centrality. Bilateral donors such as USAID (United States Assistance for International Development), the Japanese Government and the Canadian Government were also included in the study. They apparently divided their funds to various organizations ranging from local to international organizations. However, in regard to *betweenness* centrality, the bilateral donors seem to have less influence. One of the main reasons why nodes such as UNDP show such a high connection is because they play intermediary roles between donors, governments and civil society.

**Table 2. Sums of degree, outdegree and indegree:** Source: Author. Data from BRR April 2007. The calculation uses Pajek mode 1 (directed network).

Groups	Degree	Indegree	Outdegree	# of orgs	Degree (%)	Outdegree (%)	Indegree (%)	# orgs (%)
Other Organizations	0.014	0.010	0.019	19	1.2%	0.8 %	1.5%	2.4%
University	0.015	0.005	0.025	10	1.2%	0.4%	2.0%	1.3%
Private Firms	0.043	0.067	0.019	51	3.5%	5.4%	1.5%	6.4%
Local/national NGOs	0.180	0.029	0.332	147	14.7%	2.3%	27.0%	18.4%
International NGOs	0.695	0.720	0.670	472	56.5%	58.5%	54.4%	59.2%
Multilateral orgs	0.133	0.185	0.082	25	10.8%	15.0%	6.6%	3.1%
Bilateral orgs	0.101	0.200	0.001	36	8.2%	16.2%	0.1%	4.5%
Local governments	0.024	0.009	0.039	28	1.9%	0.7%	3.2%	3.5%
National govt orgs	0.012	-	0.024	8	1.0%	0.0%	1.9%	1.0%
BRR	0.011	0.004	0.018	1	0.9%	0.3%	1.4%	0.1%
Total	1.23	1.23	1.23	797	100%	100%	100%	100%

Figure 3 shows that multilateral organizations comprised only 3.1% (25 organizations) of the total organisation, but they enjoyed a higher percentage in outdegree (15%). Bilateral donors comprised 4.5% (36 countries – as registered in the April 2007 database), however, their *outdegree* was 16.2%. BRR as the reconstruction authority is only 1 out of 797 (or 0.1%) but its *indegree* is 1.4% (which is quite high and shows its level of importance as the highest reconstruction authority). Overall, in terms of relational ties, INGOs have the higher percentage of degree distribution (56.5), slightly lower than their total number (59.2% or 172 organizations). Local NGOs ranked second.

This analysis demonstrates some interesting results. Bilateral organizations tend to play roles as donors. They tend to have high outdegree (Figure 3), but very low *indegree* (Table 2). This confirms the reality that donors are the ones that give grants, not receive grants. However, the reason why donors’ *indegree* is not zero is due to the existence of intermediary donors (or grant-­making organizations that receive money from other organizations). Local NGOs most often played roles as recipients of funds – proved by low *outdegree* but high *indegree*. Multilateral organizations (such as United Nations agencies) tend to play roles as both a grantor and a grantee. International NGOs tend to play the same roles as multilateral organizations, as they are grantees as well as grantors. Some private firms (such as commercial banks) played roles as grantors for the Indian Ocean Tsunami 2004, and some local firms become project implementers. Meanwhile, BRR played both roles: as a grantee in order to be the grantor.

**Degree, indegree and outdegree distribution analysis:**
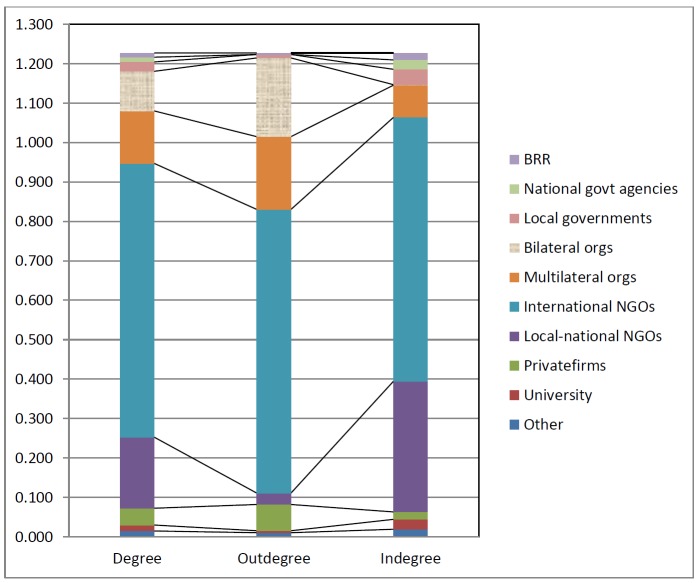
*Degree, outdegree* and *indegree* based on the categories of actors. Calculation based on Pajek.

**Degree and Betweenness Centrality distrbution:**
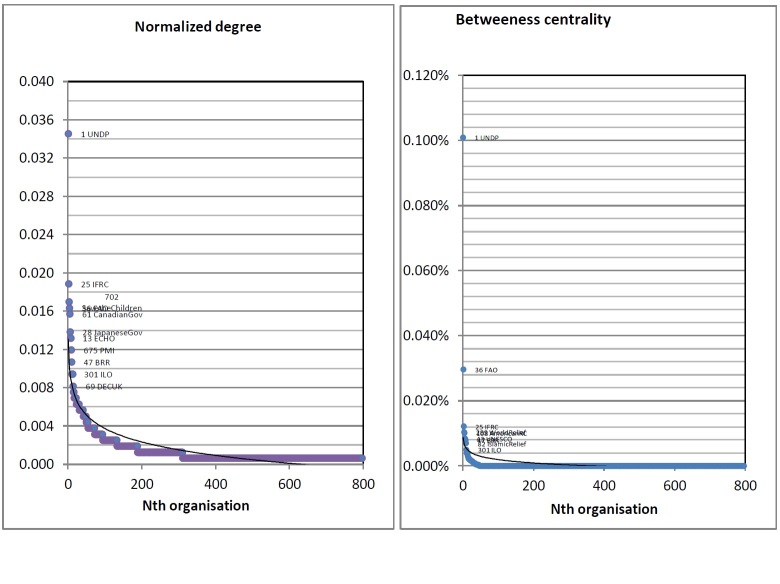
Calculation was based on Pajek’s algorithm *Betweenness *and* degree* centrality.

Figure 4 shows the ‘power law’ phenomenon as seen (*degree* distribution and *betweenness* centrality distribution). The United Nations Office for Coordination and Humanitarian Affairs (OCHA), under the United Nations system, is the mandated organization responsible for major humanitarian coordination and reconstruction coordination and information management, and is not included as a ‘leader’ as measured by *betweenness* centrality. One of the reasons is that this exercise is based on the BRR database on reconstruction for “who does what supported by whom” and it does not reflect the whole picture of how a coordinating agency played roles in the field. The reason BRR is part of the top 10 leaders in the selected network is due to its role as a donor in the reconstruction process (see Figure 4). Based on a heuristic or an educated guess, one may not be surprised with the FAO and IFRC being on top, together with the UNDP. The question is whether this measure of financial transaction is the best way to understand coordination. The answer is it is not the only way to measure coordination as long as there is other data available to suggest more detail analysis.

**Table 3. Shrinked network:** Source: Author. Data from BRR April 2007. The calculation uses Pajek mode 1 (directed network).

Shrinked networks	# of nodes	% of nodes	# of links	% of links
2-core	249	31.05	478	48.93
3-core	76	9.48	186	19.04
≥ 5-degree nodes	92	11.47	160	16.38
≥ 10-degree nodes	29	3.62	42	4.03
≥ 15-degree nodes	13	1.62	17	1.74

However, in Social Network Analysis, there is already established knowledge concerning the strength of small ties that may be shadowed by the large connection of some nodes, which may be missed by a non­-SNA expert. The concept of the ‘strength of small ties’ is already common and can be found in the Nooy, Mrvar, and Batagelj.^22^ What is also interesting is the fact that *betweenness* centrality measures the ‘true’ leaders on the ground, which brought some unfamiliar (especially before the 2004 Indian Ocean tsunami) names to the humanitarian network in Indonesia – such as Tearfund UK, World Relief, American Red Cross, Mercy Malaysia, and so on. The result is rather counter-intuitive but indeed important for the government and preexisting national network to recognize the emerging important actors on the field, for better humanitarian coordination.

## 6. Discussion

**Insights for Network and Social Network Theory**

It is quite surprising that the diameter of humanitarian organizations is 5. Take any two organizations, of which one is any local NGO and the other is any international NGO, and the findings suggest that either the former or the latter will need, on average, five intermediaries to get connected for transaction. This suggests that humanitarian actors’ network typology in the context of large catastrophic disasters in the developing world (like Aceh, Indonesia) reflects real-­world individual networks, as shown by former studies such as the work by Milgram.^5^^,^ 25 Milgram conducted an experiment where he targeted two persons in Boston by sending 160 letters from Wichita, Kansas, and Omaha, Nebraska. His objective was to know how many links or ‘intermediate persons’ were needed for any two people. Based on this experiment in the US, Milgram (1967)^25^ found that the distance between any two people is connected by 5.2 ‘intermediate persons’, or ‘degrees of separation’. Dodds, Muhamad and Watts experimented with 60,000 email users in an attempt to reach one of 18 target persons in 13 countries by forwarding messages to acquaintances, which involved 24,163 email chains; only 1.59% reached the targets. Their network is a ‘small­-world network’ which concludes that “social searches can reach their targets in a median of five to seven steps, depending on the separation of source and target, although small variations in chain lengths and participation rates generate large differences in target reachability.”^26^

The measure of network diameter is important because it shows the maximum distance between any two disaster response organizations. The implication of the network diameter in times of emergency intervention is more serious than Milgram’s ordinary social network. It is about life and death decisions and where organizations should get connected to achieve their common goals in saving lives and rebuilding the livelihoods of survivors. This means that if high authoritative agencies such as the

reconstruction authority BRR and United Nations Office for Coordination of Humanitarian Affairs (OCHA) were willing to ensure level of quality control for a thousand organizations, they could simply send emails to all of them. However, how could they get the addresses or emails of those organizations? It may seem obvious that by reaching through the humanitarian clusters, they could reach the other organizations that were partners of the cluster members. The thing is, how can the non­cluster actors be connected? Reaching out to all the actors is obviously a heavy task. One can argue that the authorities can simply use other forms of media. However, the realities on the ground are not that simple. The author argues that the flow of technical knowledge that ensures quality of implementation often flows according to the flow of grants. Implementing partners and aid distributors to communities tend to only comply with their funders. The intention to avoid overlaps of aid cannot be fully controlled along the almost 1000 kilometers of affected coastal communities (from the Nias Islands to south of Aceh to the west of Aceh and to the far east of Aceh).

This research shows the *degree* distribution follows power law due to the ‘preferential attachment’ phenomenon,^27^ where some of the most highly connected nodes are those that are the leads of humanitarian clusters. More important, the cluster leads often play intermediary roles that connect NGOs, governments, donors and private sectors. The birth of new NGOs after disasters is likely to be connected to certain highly connected nodes. The implication of this finding is that for other large-­scale disasters in the developing world, such as Haiti, Myanmar and Pakistan, the network’s structure is more than likely the same. This begs for more investigation and research.

What is unique about this research is the fact that it is not an experimental research. It is based on the real records concerning 1300 financial updates from almost 800 different organizations. Even though it does not reflect the absolute number of humanitarian and reconstruction organizations in Aceh during 2005­/2007, the recorded list is estimated to be more than half of the total actors. In addition, all of these international and national actors were more or less used to visiting or to being based for a certain period of time in Aceh Province and the Nias Island during 2005­/2007.

The question remains whether all of the links between the nodes can only be explained by financial transaction. The answer is, of course, not necessarily. Email communications can be one of the options. However, getting all the email records from the actors is also a serious challenge. The most important steps in network analysis are clearly defining what are the nodes and the links represented. In this exercise, the links are the financial transactions. The nodes are the organizations. Therefore, for future research, one could investigate more complex dimensions where the nodes can be any organization and any individual and the links can either be more broadly defined (financial transactions, knowledge and innovation sharing and standards) or more specific in relations, such as informal gatherings of individuals.

**Insights of network theory for disasters studies**

The findings have significant implications for disaster management communities. Field coordination of humanitarian emergency actors is a complex and difficult task. The author did not expect to find that the network typology of humanitarian and post­-disaster reconstruction actors is similar to real world social networks.^25^^,^ 26 Despite critics’ concerns over Milgram’s^25^ incomplete chain of letters to the targeted subject, the incomplete chain of letters reflects the real world difficulties of doing ‘coordination’ and the problems of policy coordination in real, chaotic disaster situations.

The use of Aceh’s reconstruction updates provides more realistic views of the organizational coordination. It is also noted that the emergence of hubs in humanitarian networks, namely humanitarian clusters, are proven to be central nodes. Therefore, governing post-­disaster interventions can be better guided by understanding this phenomenon. United Nations agencies and local authorities can improve coordination effectiveness through the existing humanitarian clusters. What is lacking is that some hubs are not included in the (traditional) humanitarian clusters. Therefore, the vision of coordination should move beyond the existing humanitarian clusters. Ramalingam et al. highlights that cross­-organizational networks have played pivotal roles in post­-disaster interventions in recent decades.^25^^,^ 28

When a disaster emergency occurs at the scale of, or larger than, the 2004 Indian Ocean tsunami, there can suddenly develop an ad-­hoc big­-bang formation of humanitarian emergency networks. The networks often grew and then faded away. Furthermore, they may be transformed into new network structures. Key government agencies were often not able to comprehend the complexity, and the network novelty grows as thousands of events (intervention projects) occur during the emergency and reconstruction phases. The emergency network may later transform into a new network as new reconstruction and recovery begins in a new disaster ­affected area in another part of the world.

Large-­scale disasters in developing countries triggered more than a hundred donor countries, hundreds of international NGOs that also serve as donors, and created the new formation of local NGOs in Aceh and Sri Lanka; Cyclone Nargis in Myanmar, a devastating earthquake in Haiti, and floods in Pakistan 2010 led to the recruitment of thousands reconstruction workers from hundreds of NGOs.

This analysis can be done as the events (or humanitarian responses) occur on the ground. It may create opportunities for respective authorities to play smart coordination roles through several informed decentralized systems. Organizations like OCHA have often played roles in the first week of disasters in developing worlds like Indonesia. Their approach, to document “who is doing what where and when”, can be rapidly analyzed regularly in the field. However, this requires human resources which are often not locally available. Nevertheless, as long as there is accurate information concerning “who is doing what where and when” and as long as there is qualified staff at headquarters, the analysis can be done and networks can be monitored regularly. In addition, if this can be done, the formation of a network and the burst of the network can be adequately monitored before, during and after humanitarian mission.

There is confirmed evidence that post-­disaster intervention after the 2004 Indian Ocean tsunami emerged as a governance network. The involvement of actors and stakeholders (from the local to the global level) ranged from local NGOs, to national and local governments, to international financial institutions and the United Nations, to universities, private firms, bilateral aid and so on. Government is not the only central actor, as there are many central actors as evidenced by the centrality analysis (*degree* and *betweenness*). This confirms both the theory and the hypothesis that post­-disaster governance emerges as a polycentric network with many centers of authority that devise responsibility for post-­disaster intervention.

The exercise can go beyond the grantors-­grantees relationship as presented in this paper. Real exercises on the ground should be possible, and network theory can help coordinating agencies, such as disaster risk management authorities (local and national) and international humanitarian coordinating agencies such as OCHA, and other humanitarian clusters’ leaders to map the complex landscape of post-­disaster intervention in order to inform their actions to provide more effective and efficient intervention. Based on the experience from Aceh, the author also suggests that the concept of humanitarian cluster approaches can be strengthen using social network analysis. This can certainly help both national and international intervention systems to be more effective and efficient.

The emergence of hubs highlights the strength of a disaster governance framework, because the hubs are in fact ‘multiple centers’ where command and resources are flowed through to the fields. This is the ‘poly­centric’ feature of emergency and reconstruction management. It promotes the notion that there are many overlapping centers of authority and responsibility for disaster ­risk reduction and post­-disaster intervention. It can be concluded that the structure of a post­-disaster system is highly decentralized. Therefore, any effort to guarantee the quality of interventions must understand the nature of the network. This phenomenon is called ‘networked governance’ of post­-disaster interventions.

## 7. Conclusion

Large-­scale disaster risks bring their own typology of actors’ networks. However, the network is not randomly formed. Interestingly, the network diameter reflects the real world network. This seems to be counter intuitive, as people may think that the level of ties or connection between any two humanitarian actors in a specific disaster ­affected geography can be less than real ­world individual networks. It is clear that without understanding the landscape of complexity, government authority may not be able to create ‘organized behavior’ among nearly thousands of reconstruction players to guaranty quality in emergency intervention and reconstruction.

There are limitations in this research. Despite clear operational benefits of this approach, future works should provide more empirical evidence from recent large-­scale disasters beyond financial transaction. This analysis is limited to ‘principal-­client’ networks among donors and implementers, regardless of the localities where they work. More exploration on the different use of social network analytical tools for disaster studies is suggested. Cases from Haiti can also be presented in the future (work in progress). The application of the theory is arguably wide and can be applied in the wider context of disaster research. This includes more valuable measurements such as the density of a network that can be measured over different periods of time (rather than treating the network as a single period).

Post-­disaster governance is therefore not entirely unique. It is rather a micro­cosmos of real world networks. However, more comprehensive study concerning the type and scale of disasters and their typical networks may guide authorities in the United Nations and governments to perform better in future post-­disaster interventions.

## Competing Interest Declaration

The author has declared that no competing interests exist. This research is an independent work.
